# Truncated mutants of beta-glucosidase 2 (GBA2) are localized in the mitochondrial matrix and cause mitochondrial fragmentation

**DOI:** 10.1371/journal.pone.0233856

**Published:** 2020-06-03

**Authors:** Saki Sultana, Jacklyn Stewart, Aarnoud C. van der Spoel

**Affiliations:** 1 The Atlantic Research Centre, Department of Biochemistry & Molecular Biology, Dalhousie University, Halifax, Nova Scotia, Canada; 2 Biomedical Sciences Program, Dalhousie University, Halifax, Nova Scotia, Canada; 3 The Atlantic Research Centre, Department of Pediatrics, Dalhousie University, Halifax, Nova Scotia, Canada; University of Waterloo, CANADA

## Abstract

The enzyme β-glucosidase 2 (GBA2) is clinically relevant because it is targeted by the drug miglustat (Zavesca^®^) and because it is involved in inherited diseases. Mutations in the *GBA2* gene are associated with two neurological diseases on the ataxia-spasticity spectrum, hereditary spastic paraplegia 46 (SPG46) and Marinesco-Sjögren-like syndrome (MSS). To establish how *GBA2* mutations give rise to neurological pathology, we have begun to investigate mutant forms of GBA2 encoded by disease-associated *GBA2* alleles. Previously, we found that five GBA2 missense mutants and five C-terminally truncated mutants lacked enzyme activity. Here we have examined the cellular locations of wild-type (WT) and mutant forms of GBA2 by confocal and electron microscopy, using transfected cells. Similar to GBA2-WT, the D594H and M510Vfs*17 GBA2 mutants were located at the plasma membrane, whereas the C-terminally truncated mutants terminating after amino acids 233 and 339 (GBA2-233 and -339) were present in the mitochondrial matrix, induced mitochondrial fragmentation and loss of mitochondrial transmembrane potential. Deletional mutagenesis indicated that residues 161–200 are critical for the mitochondrial fragmentation of GBA2-233 and -339. Considering that the mitochondrial fragmentation induced by GBA2-233 and -339 is consistently accompanied by their localization to the mitochondrial matrix, our deletional analysis raises the possibility that that GBA2 residues 161–200 harbor an internal targeting sequence for transport to the mitochondrial matrix. Altogether, our work provides new insights into the behaviour of GBA2-WT and disease-associated forms of GBA2.

## Introduction

The enzyme β-glucosidase 2 (GBA2) cleaves glucose off the sphingolipid glucosylceramide and related compounds [1–5] and can also transfer glucose and galactose from glucosylceramide and galactosylceramide, respectively, to cholesterol [6–8]. Thus far, limited insights into the physiological role of GBA2 have been obtained by pharmacologically inhibiting the enzyme, by gene ablation, and through the identification of mutations in the *GBA2* gene in humans affected with neurological diseases.

In mice, administration of the GBA2 inhibitor miglustat and disruption of the *Gba2* gene elevate the glucosylceramide level in testis, spleen, and brain [4, 5] and impair spermatogenesis [9–11], resulting in male infertility [5, 12, 13]. Notably, the reproductive effect of miglustat was only observed in five of the 18 distinct inbred mouse strains evaluated for this trait [14, 15]; the five miglustat-sensitive strains all belong to the C57-family of inbred strains. In humans, pharmacological inhibition of GBA2 does not affect spermatogenesis [16]. Further, long-term miglustat administration causing a five-fold increase in brain glucosylceramide [4] does not affect motor co-ordination, muscle strength or exploratory behaviour of C57BL/6 mice [12]. Disruption of the *Gba2* gene, however, affects locomotion, altering the step pattern [17].

Mutations in the human *GBA2* gene result in diseases on the ataxia-spasticity spectrum, cerebellar ataxia/hereditary spastic paraplegia 46 (SPG46) [18–27] and Marinesco-Sjögren-like syndrome (MSS) [28]. SPG46 has an early childhood onset, and disease manifestations include muscle weakness and spasticity in upper and lower limbs, ataxia, axonal neuropathy, cognitive impairment, thin corpus callosum, and cerebellar and cerebral atrophy [18, 21, 23, 25]. No adequate therapies are available for SPG46 or for most other forms of hereditary ataxias and paraplegias.

Altogether, targeting GBA2 does not have the same consequences in humans as in mice; humans depend to a much greater extent on GBA2 for proper development of the central nervous system and motor control in arms and legs. This species difference is not understood at present [29]. Further, it may appear paradoxical that, in humans, different ways of targeting GBA2 have different consequences, as the GBA2 inhibitor miglustat is well tolerated while mutations in the *GBA2* gene result in a range of neurological and non-neurological symptoms. However, these two ways to target GBA2 differ greatly in their timing; mutations in *GBA2* are present from the moment of fertilization, while miglustat is administered much later in life. This would suggest that GBA2 has a critical role early in life and is less important in adulthood.

**Fig 1:**
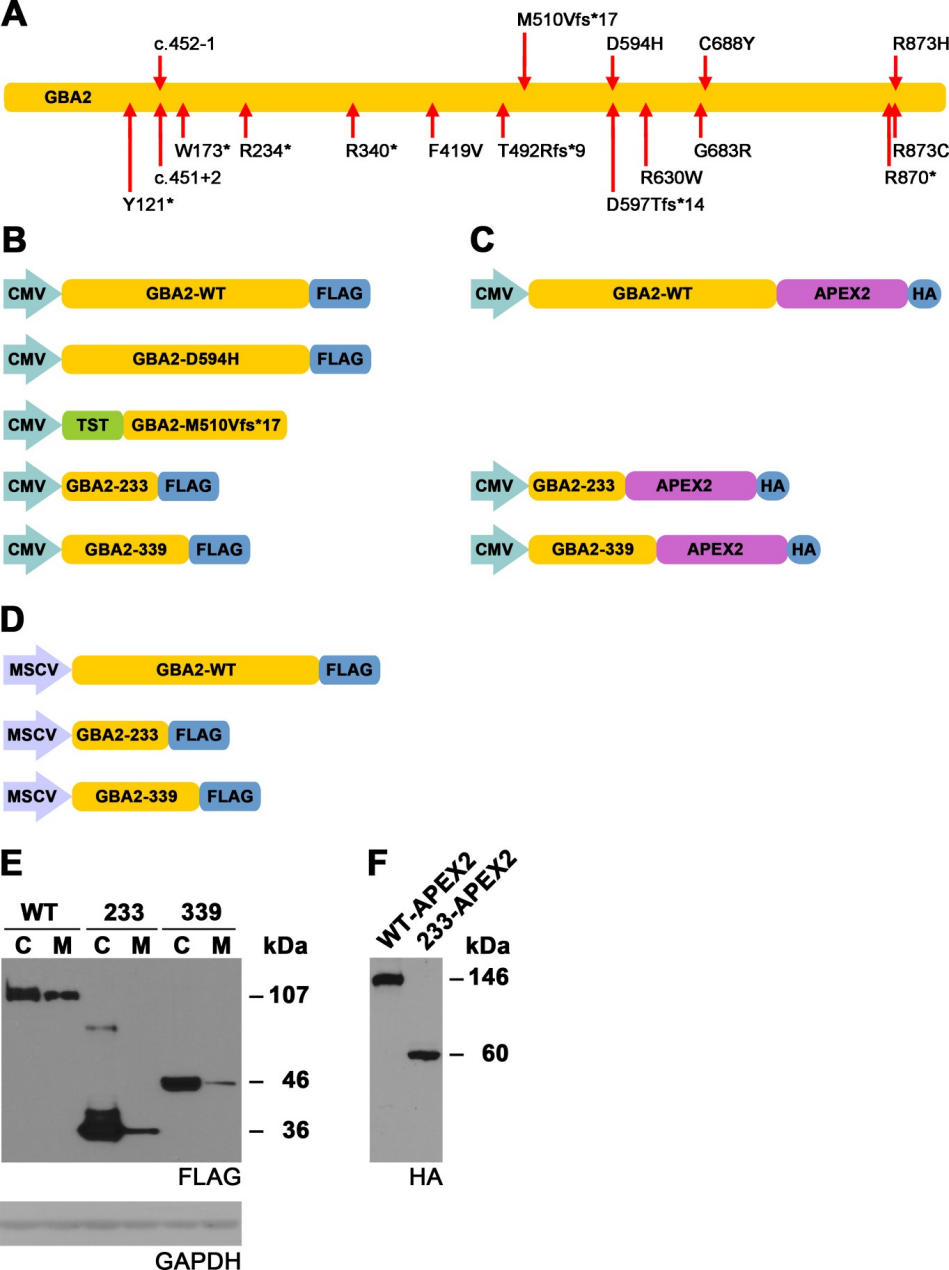
Expression of GBA2-WT and mutant forms of GBA2. (A) Schematic overview of mutations identified in the *GBA2* gene in hereditary spastic paraplegia 46 (SPG46) [18–27] and Marinesco-Sjögren-like syndrome (M510Vfs*17) [28]. Indicated are the consequences of the *GBA2* mutations at the amino acid level, except for two intronic mutations for which the nucleotide changes (c.) are indicated. (B) Schematics of the constructs used in this study for the expression of various forms of GBA2 under the control of the CMV promoter: GBA2-WT, the missense mutant D594H, and the nonsense mutants terminating at residues 233 and 339 (GBA2-233 and GBA2-339), all carrying a C-terminal FLAG tag, and the frameshift mutant M510Vfs*17 carrying an N-terminal twin-strep-tag (TST). (C) Schematics of the constructs used in this study for the expression of C-terminally APEX2-tagged forms of GBA2-WT, GBA2-233, and GBA2-339, under the control of the CMV promoter. (D) Schematics of the constructs used in this study for the expression various C-terminally FLAG-tagged forms of GBA2 under the control of the MSCV LTR: GBA2-WT, GBA2-233, and GBA2-339. Note: schematics in [B], [C], and [D] are not drawn to scale. (E) Western blot showing the relative levels of GBA2-WT-FLAG, GBA2-233-FLAG, and GBA2-339-FLAG in U2OS cells under different promoters; C, CMV promoter; M, MSCV LTR. (F) Western blot showing the expression of APEX2-HA-tagged forms of GBA2-WT and GBA2-233; apparent molecular weights are indicated.

The genetic abnormalities identified in the human *GBA2* gene include missense, nonsense, splice site, and frameshift mutations (Fig 1A), most of which are present in a homozygous fashion. It is unclear how the mutations in the *GBA2* gene give rise to the range of disease manifestations seen in SPG46 and MSS patients [29]. To obtain insights into the physiological role of GBA2 and into the etiology of SPG46 and MSS, we previously modeled ten SPG46-associated *GBA2* mutations in the cDNA coding for human GBA2, and found that single amino acid-substituted and C-terminally truncated forms of GBA2 failed to cleave its substrate [22]. Here, we have focused on wild-type GBA2 (GBA2-WT) and on three forms of GBA2 that are encoded by SPG46-associated *GBA2* alleles, the single amino acid-substituted D594H mutant (c.1780G>C) [23] and the C-terminally truncated mutants terminating after amino acids 233 (c.700C>T, p.R234*) [21] and 339 (c.1018C>T, p.R340*) [18], respectively. We have also included in our study the form of GBA2 encoded by the MSS-associated *GBA2* allele, the C-terminally truncated frameshift M510Vfs*17 mutant (c.1528_1529del) [28]. Using electron microscopy, we found that GBA2-WT is located at the plasma membrane. We have also established that GBA2-233 and GBA2-339 are efficiently imported into the mitochondrial matrix, cause mitochondria to lose their transmembrane potential, and convert mitochondrial tubules to punctate structures. Our study provides new insights into the cell biology of wild-type and mutant forms of human GBA2-WT. Parts of this work have previously been published in abstract form [30].

## Materials and methods

### Cell culture

U2OS, SH-SY5Y, and HeLa cells (ATCC) were maintained in DMEM supplemented with 10% fetal calf serum. Primary rat hippocampal cells were obtained and cultured as described [31]. Animal use for this purpose was approved by the Dalhousie University Committee on Laboratory Animals (approval number 19–108). Rats were sacrificed by anesthesia with isofluorane (5% v/v) followed by CO2 asphyxiation. Hippocampi were dissected from E18 Sprague-Dawley rat embryos, incubated with 0.03% trypsin for 15 minutes, and dissociated using a fire-polished Pasteur pipette. Cells were then plated on coverslips coated with 0.1% (w/v) poly-L-lysine (Peptides International) at a density of 3-6x10^3^ cm^-1^ in Neurobasal Medium (Invitrogen) supplemented with 2% B-27 (Invitrogen), 50 μM glutamine, 25 μM glutamate, 5% fetal calf serum, 100 U/ml penicillin, and 100 μg/ml streptomycin. After 4 hours of plating, the medium was replaced with serum-free Neurobasal Medium supplemented with B-27 and 0.5 mM glutamine. One-third of the medium was replaced on a weekly basis until transfection.

### DNA constructs

cDNAs encoding GBA2-WT, GBA2-233, GBA-339, and GBA2-D594H were described previously [22]. To generate vectors allowing low-level expression of various forms of GBA2, cDNAs encoding C-terminally FLAG-tagged forms of GBA2-WT, GBA2-233, and GBA-339 were subcloned into BglII-KspAI-digested pMSCV-puro-PAX9 (Addgene #75079; a gift from David Mu [32]).

cDNAs encoding additional forms of GBA2, twin-strep-tag(TST)-GBA2-M510Vfs*17, and APEX2-tagged forms, constructs containing a T2A sequence, and N-terminal deletions of GBA2-339 were generated by inserting or replacing portions of the original GBA2 cDNA and previously prepared constructs [22] with synthetic DNA fragments (BioBasic, Twist Bioscience) via restriction digestion/ligation cloning and HD In-Fusion cloning (Takara). APEX2 and mClover2 coding sequences were provided by Alice Ting (www.addgene.org/49385) [33] and Michael Davidson and (www.addgene.org/54577), respectively. All new cDNA constructs were verified by double-strand Sanger sequencing.

### Transfections and western blotting

Cell lines were cultured to a subconfluent state and transfected with endotoxin-free plasmid DNA using Lipofectamine 2000 (ThermoFisher) or *Trans*IT-LT1 (Mirus) transfections reagents following manufacturers’ instructions. Two days post-transfection, cells were washed in PBS, harvested by scraping, and lysed in 20 mM Tris-HCl, 300 mM KCl, 0.5 mM EDTA, 0.5 mM EGTA, 10% glycerol, 0.25% Nonidet P-40 and protease inhibitors (cOmplete ULTRA protease inhibitor cocktail, Roche). Samples of equal total protein content were separated by SDS-PAGE and transferred to PVDF membrane by semi-dry Western blotting in 50 mM Tris-base,

40 mM glycine, 0.0375% SDS, and 20% methanol. Western blots were probed with primary antibodies raised against the FLAG-tag (mouse mAb, clone M2, Sigma F1804; 1:1000), and biotin-conjugated Anti-HA (rat mAb, clone 3F10, Roche 12158167001; 1:1000), anti-GAPDH (rabbit mAb, clone 14C10, Cell Signaling; 1:40,000), anti-α-tubulin (mouse mAb, clone DM1A, Upstate; 1:40,000 / rabbit mAb, Cell Signaling 2125S; 1:20,000). Blots were developed with HRP-conjugated streptavidin (ThermoFisher Scientific) or HRP-conjugated secondary antibodies (Jackson ImmunoResearch), and chemiluminescent substrates (Amersham ECL, GE Healthcare; SuperSignal West Pico (PLUS), ThermoFisher). Primary rat hippocampal cells were transfected 10–14 days after plating using a calcium-phosphate precipitation protocol [31].

### Immunostaining & microscopy

Cells grown on glass coverslips were washed in PBS, fixed in 4% (w/v) paraformaldehyde in PBS for 25 min, quenched 5 mM ammonium chloride in PBS (2 x 15 min), permeabilized in ice-cold 0.3% Triton X-100 in PBS for 20 min, washed in PBS (2 x 15 min), blocked in 1% BSA in PBS for 1 hr, and incubated in primary antibody diluted in PBS/BSA at 4°C for 16 hr. Rabbit anti- DYKDDDDK (FLAG) mAb was used at 1:2000 (Cell Signaling 2368S), mouse anti-TOMM20 mAb at 1:250 (BD Transduction Laboratories 612278), mouse anti-cytochrome c mAb at 1:1000 (BD Bioscience 556432), and anti-TST (StrepMAB-Classic, IBA 2-1507-001) at 1:1500. After two 15-min washes in PBS/BSA, cells were incubated in a fluorescently labeled secondary antibody (Alexa Fluor, ThermoFisher Scientific), washed in PBS/BSA, PBS, and water. Coverslips were dried to air and mounted on glass slides with Vectashield antifade medium (Vector). Cells were imaged via confocal laser scanning microscopy (Zeiss LSM 510).

To indirectly detect APEX2-tagged proteins via fluorescence microscopy, cells grown on glass coverslips were incubated with biotin-phenol, briefly exposed to hydrogen peroxide, quenched and washed as described [33], then fixed, permeabilized, and washed as described above, and incubated with Alexa594-conjugated streptavidin at 1:1000 (Invitrogen S11227).

### Electron microscopy

Electron microscopy using the APEX2 tag was performed as described [34]. At 48 hours of post-transfection, cells expressing APEX2 constructs were briefly washed with cacodylate buffer containing 2% glutaraldehyde at 37°C, fixed with cacodylate/glutaraldehyde for 1 hour on ice, washed with ice-cold 100 mM sodium cacodylate buffer, pH 7.4 (5 x 2 min), quenched with 20mM glycine in sodium cacodylate buffer (10 min), and washed again with cacodylate buffer (5 x 2 min). Cells were incubated in 0.5 mg/ml diaminobenzidine (from freshly prepared 10x stock in 100 mM HCl) and H_2_O_2_ (0.03%) in cacodylate buffer for 25 minutes, washed with cacodylate buffer, fixed with freshly prepared 2% osmium tetroxide (OsO_4_) for 30 minutes, washed with ice-cold milli-Q water several times, and incubated with 2% uranyl acetate solution overnight. Cells were gently scraped off the culture dish and pelleted, dehydrated through a graduated series of acetone, and embedded in Epon Araldite resin, which was cured at 60°C for 48 hours. Ultrathin sections were cut using a Reichert-Jung Ultracut E Ultramicrotome with a diamond knife, placed on 300 mesh copper grids which, and stained with 2% uranyl acetate and lead citrate. Sections were viewed using a JEOL JEM 1230 Transmission Electron Microscope at 80kV and imaged using a Hamamatsu ORCA-HR digital camera.

### Mitochondrial membrane potential

U2OS cells seeded on 35 mm glass-bottom culture dishes (Ibidi USA Inc., Fitchburg, WI; 100,000 cells/dish) were transfected to co-express mClover2 and different forms of GBA2. At 48 hours post-transfection, the culture medium was replaced with FluoroBrite DMEM (Gibco, Grand Island, NY) containing 10% FBS and 200 μM L-glutamine, supplemented with tetramethyl-rhodamine methyl ester (TMRM, Invitrogen, Carlsbad, CA; final concentration of 25 nM) with or without FCCP (Sigma; 10 μM). Cells were incubated for 15 minutes at 37° C and imaged using a Zeiss LSM 510 Meta confocal microscope. Green channel images (mClover2 fluorescence) were used to draw contours around individual transfected cells, which were copied to corresponding red channel images and used as regions of interest to determine integrated TMRM intensities using ImageJ software, corrected for background intensity. For each experimental condition, 30 cells were analyzed, in triplicate.

### Statistics

Quantitative data were analyzed for statistical significance by ANOVA with Tukey’s multiple comparisons test using GraphPad Prism 8.3.0 software.

## Results

**Fig 2:**
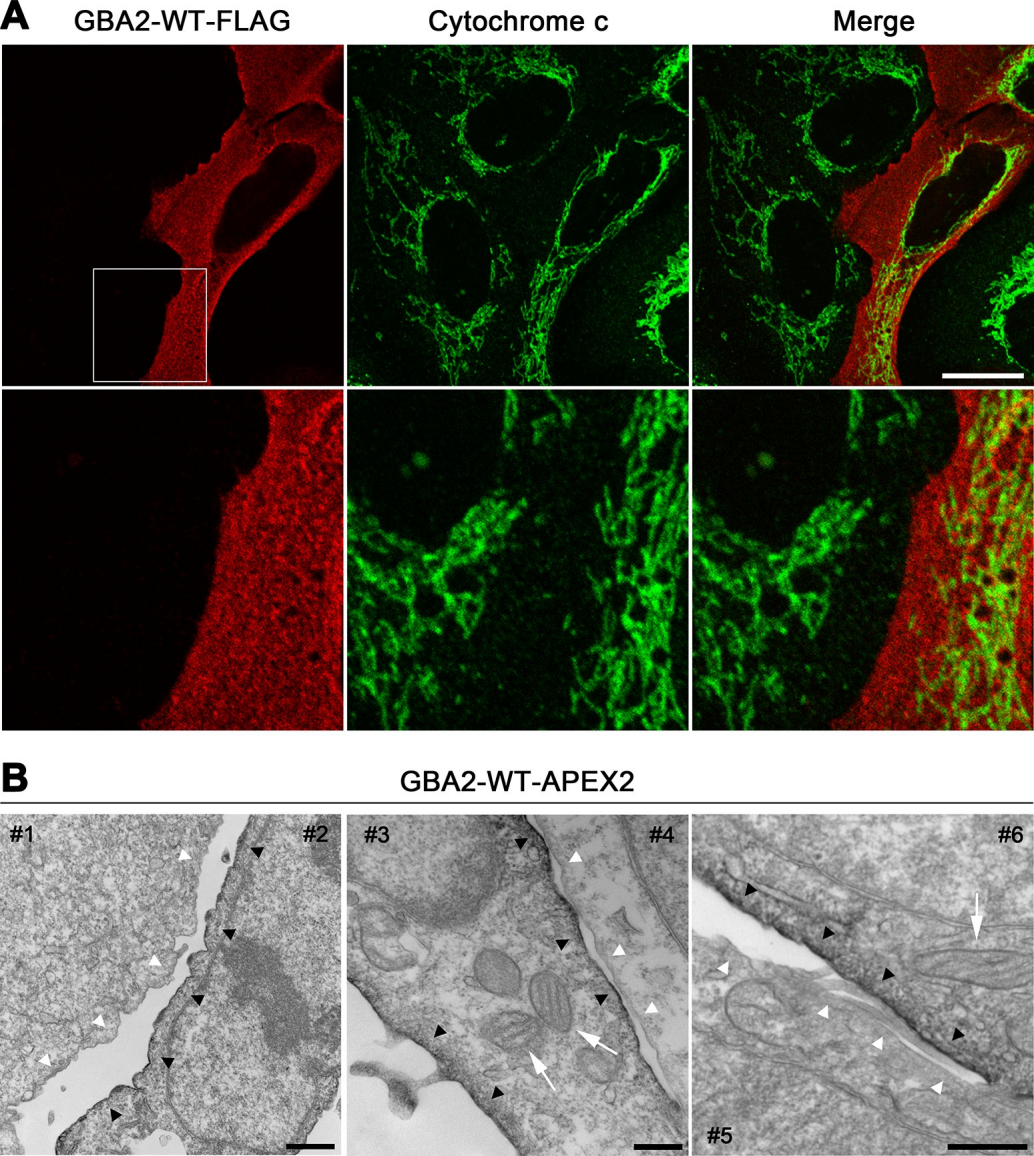
GBA2-WT is localized at the plasma membrane. (A) U2OS cells were transfected with a cDNA coding for GBA2-WT-FLAG under the control of the CMV promoter, immunostained with anti-FLAG (red) and anti-cytochrome c antibodies (green), and imaged via confocal microscopy. A section (white square) of the images in the upper panels is enlarged in the lower panels. Scale bar, 20 μm. (B) U2OS cells were transfected with a cDNA coding for GBA2-WT-APEX2 under the control of the CMV promoter, stained with DAB, and processed for electron microscopy. Each panel shows one transfected cell (#2, 3, and 6) and one non-transfected cell (#1, 4, and 5). Black arrowheads point to APEX2-stained plasma membranes; white arrowheads point to non-APEX2-stained plasma membranes. APEX2-stained plasma membranes were more electron-dense compared to non-APEX2-stained plasma membranes. White arrows point to mitochondria. Representative images are shown. Scale bars: left panel, 1000 nm; middle panel, 400 nm; right panel, 500 nm.

**Fig 3:**
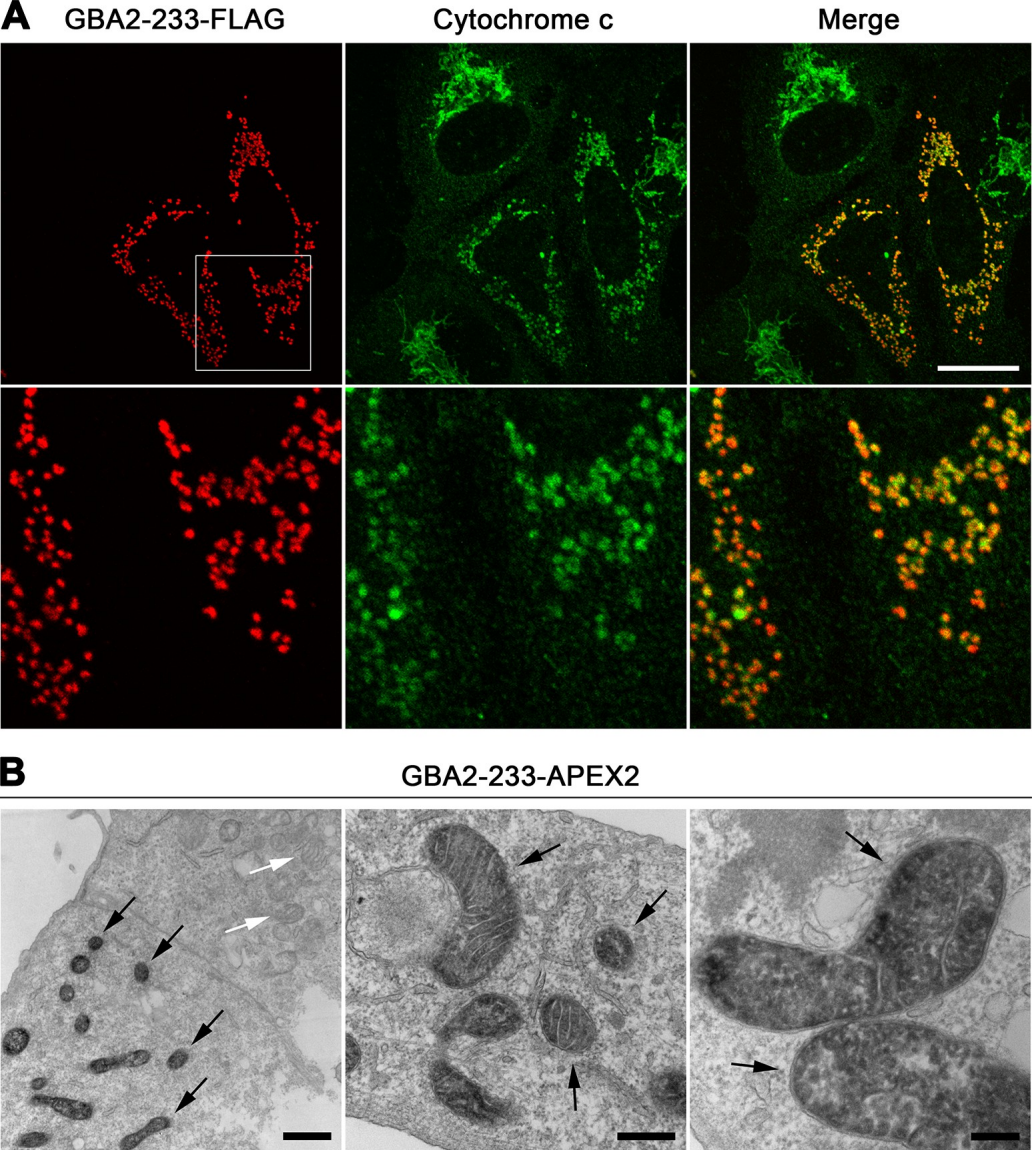
GBA2-233 is localized in the mitochondrial matrix and promotes mitochondrial fragmentation. (A) U2OS cells were transfected with a cDNA coding for GBA2-233-FLAG under the control of the CMV promoter, and immunostained with anti-FLAG (red) and anti-cytochrome c antibodies (green). A section (white square) of the images in the upper panels is enlarged in the lower panels. Scale bar, 20 μm. (B) Localization of APEX2-tagged GBA2-233 by electron microscopy. Black arrows point to APEX2-stained mitochondria; white arrows point to non-APEX2-stained mitochondria. Scale bars: left panel, 1000 nm; middle panel, 400 nm; right panel, 200 nm.

To extend our previous characterization of wild-type and mutant forms of GBA2 [22], we compared the cellular location of GBA2-WT with those of the missense D594H mutant, the frameshift M510Vfs*17 mutant, and the C-terminally truncated mutants terminating at residues 233 and 339 (GBA2-233, and GBA2-339) (Fig 1A). We expressed FLAG-tagged GBA2-WT under the control of the cytomegalovirus immediate-early promoter (CMV; Fig 1B) in HeLa, SH-SY5Y, and primary rat hippocampal cells, and in U2OS cells, and detected the protein using an anti-FLAG antibody via western blotting (Fig 1E) and immunostaining. GBA2-WT-FLAG was widely distributed throughout the cell in HeLa, SH-SY5Y, and primary rat hippocampal cells (S1A Fig, S1B Fig and S1C Fig) and in U2OS cells (Fig 2A). In another approach to localize GBA2-WT, we used the APEX2 tag (Fig 1C), which is an engineered form of soybean ascorbate peroxidase [33]. APEX2-tagged proteins traffic to many different cellular locations, depending on the protein that carries the APEX2 tag, and are transported to the same locations as the endogenous versions of these proteins [34]. APEX2 biotinylates nearby proteins when activated with hydrogen peroxide and provided with biotin-phenol [33]. Biotinylated proteins can then be detected with fluorescently labeled streptavidin. Following this approach, GBA2-WT-APEX2 (Fig 1F) displayed a wide distribution (S2A Fig), similar to GBA2-WT-FLAG (Fig 2A). APEX2 also can convert diaminobenzidine (DAB) into an insoluble osmiophilic polymer that can be directly observed via electron microscopy [34]. Upon being oxidized by APEX2, DAB remains tightly localized to its site of production [35]. We expressed APEX2-tagged GBA2-WT and exposed the cells to diaminobenzidine. GBA2-WT-APEX2 stained the plasma membrane (Fig 2B). Cells expressing GBA2-WT-FLAG and GBA2-WT-APEX2 displayed mitochondrial networks containing tubules of various lengths that closely resembled those of non-transfected cells (S1 Fig and Fig 2A). Mitochondria of non-transfected U2OS cells are also shown in Figs 3A, 5A, 6D, 8A and 8B, S3A Fig and S3B Fig, and S4A Fig and S4B Fig.

### GBA2 mutants

Similar to GBA2-WT, the D594H and M510Vfs*17 mutants were present throughout the cell (S3 Fig). By contrast, GBA2-233 was present in puncta that overlapped with a mitochondrial marker in HeLa, SH-SY5Y, and primary rat hippocampal cells (S1B Fig, S1D Fig and S1F Fig) and in U2OS cells (Fig 3A); occasionally, the punctate pattern of GBA2-233 was accompanied by an overall staining. As U2OS cells are very flat and wide, and their mitochondria lie in one focal plane without overlapping much, we used this cell line to further investigate the impact of the truncated GBA2 mutants of mitochondria.

**Fig 4:**
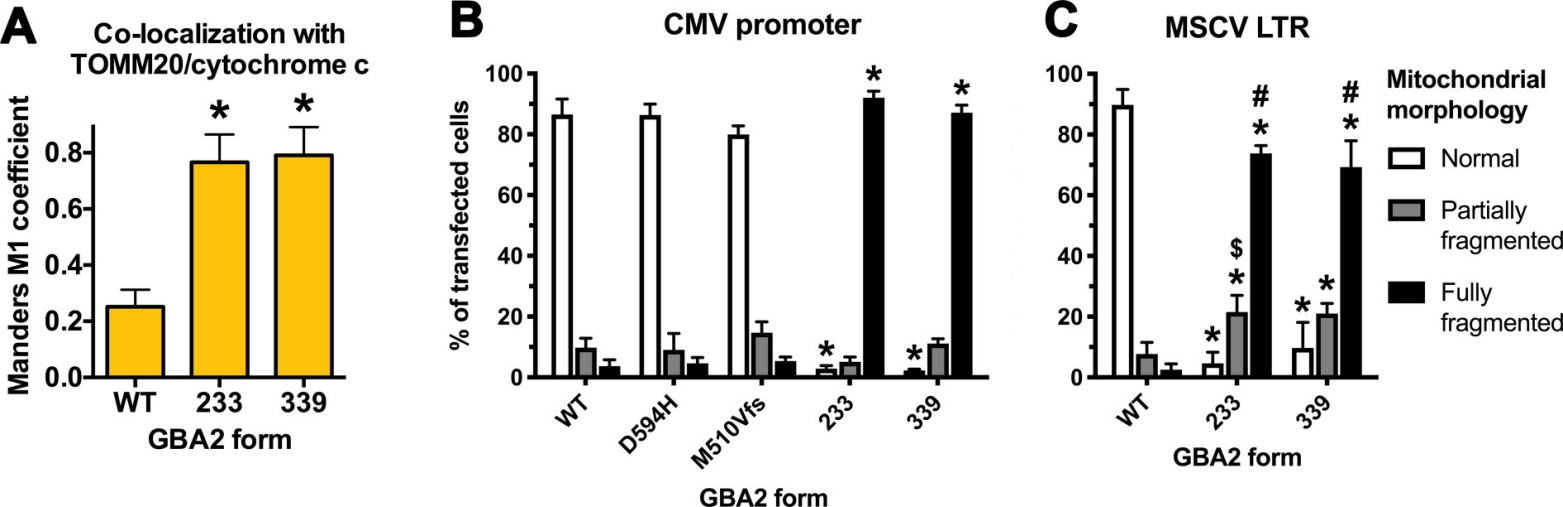
Truncated GBA2 mutants co-localize with mitochondria and induce mitochondrial fragmentation. (A) Colocalization of various forms of GBA2 with mitochondrial markers TOMM20 or cytochrome c, expressed as the Manders M1 coefficient (data indicate means + SD; n = 3; 10 cells were analyzed per experiment). (B and C) Distribution of mitochondrial morphologies among cells expressing various forms of GBA2 under the control of (B) the CMV promoter and (C) the MSCV LTR. Data indicate means + SD; at least 250 cells were scored in each of three independent experiments. Statistical comparisons between GBA2-WT and GBA2 mutants by (A) one- or (B and C) two-way ANOVA: *, P < 0.0001. Statistical comparisons between the same forms of GBA2 expressed under different promoters (CMV v MSCV): $, P = 0.0002; #, P < 0.0001.

**Fig 5:**
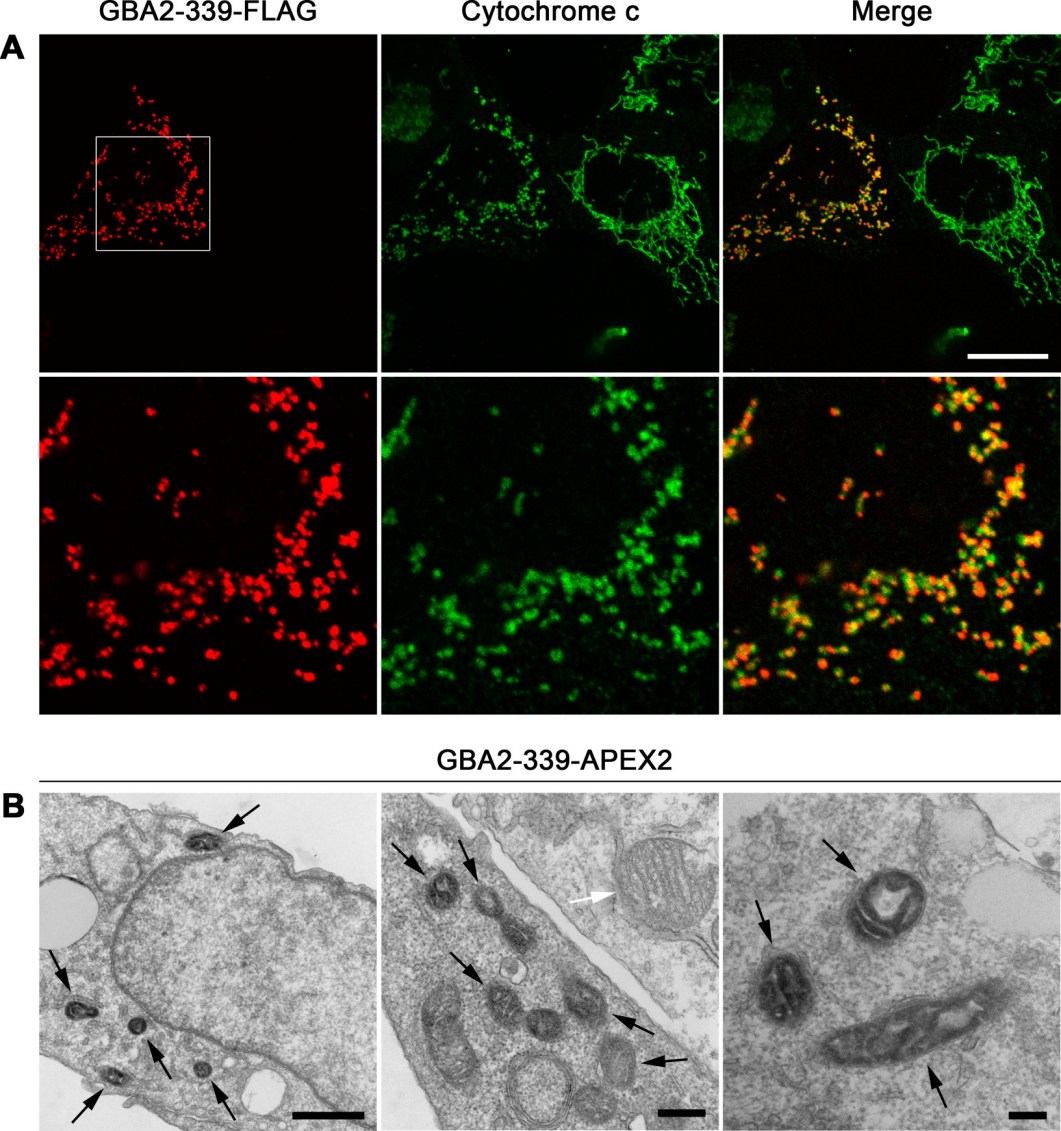
GBA2-339 is localized in the mitochondrial matrix and promotes mitochondrial fragmentation. (A) U2OS cells transfected with a cDNA coding for GBA2-339-FLAG were immunostained with anti-FLAG (red) and anti-cytochrome c antibodies (green). A section (white square) of the images in the upper panels is enlarged in the lower panels. Scale bar, 20 μm. (B) Localization of APEX2-tagged GBA2-339 by electron microscopy. Black arrows point to APEX2-stained mitochondria, and the white arrow points to a non-APEX2-stained mitochondrion. Scale bars: left panel, 1000 nm; middle panel, 400 nm; right panel, 200 nm.

GBA2-233 colocalized to a much higher degree with mitochondria compared to GBA2-WT (Fig 4A). Also, the majority of cells expressing GBA2-233 displayed very short mitochondria appearing as puncta of various sizes (Fig 3A). GBA2-233-APEX2 (Fig 1C and 1F) also localized to mitochondria with a fragmented phenotype, as detected with fluorescently labeled streptavidin (S2B Fig). At the ultrastructural level, GBA2-233-APEX2 stained the mitochondrial matrix (Fig 3B). To establish whether GBA2-233 was imported into mitochondria and altered mitochondrial morphology because of its high expression level, we expressed this mutant at a lower level using the mouse stem cell virus long-terminal repeat (MSCV LTR) as promoter (Fig 1D and 1E). Under these conditions, GBA2-233 was also present in mitochondria and caused mitochondrial fragmentation (S4B Fig), albeit in a smaller proportion of transfected cells compared to expression driven by the CMV promoter (Fig 4B and 4C). Likewise, the SPG46-associated GBA2 mutant terminating at residue 339 (GBA2-339, Fig 1A and 1B) co-localized to a high degree with mitochondria (Figs 4A and 5A), was present in the mitochondrial matrix (Fig 5B), and caused mitochondrial fragmentation in the majority of cells, both when expressed at higher and lower levels (Fig 4B and 4C). Further, our results show that the mitochondrial localization of GBA2-233 and GBA2-339 and the corresponding mitochondrial fragmentation is independent of their C-terminal tags, as FLAG-tagged and APEX2-tagged forms of these proteins behave in a similar fashion.

### Mitochondrial transmembrane potential

**Fig 6:**
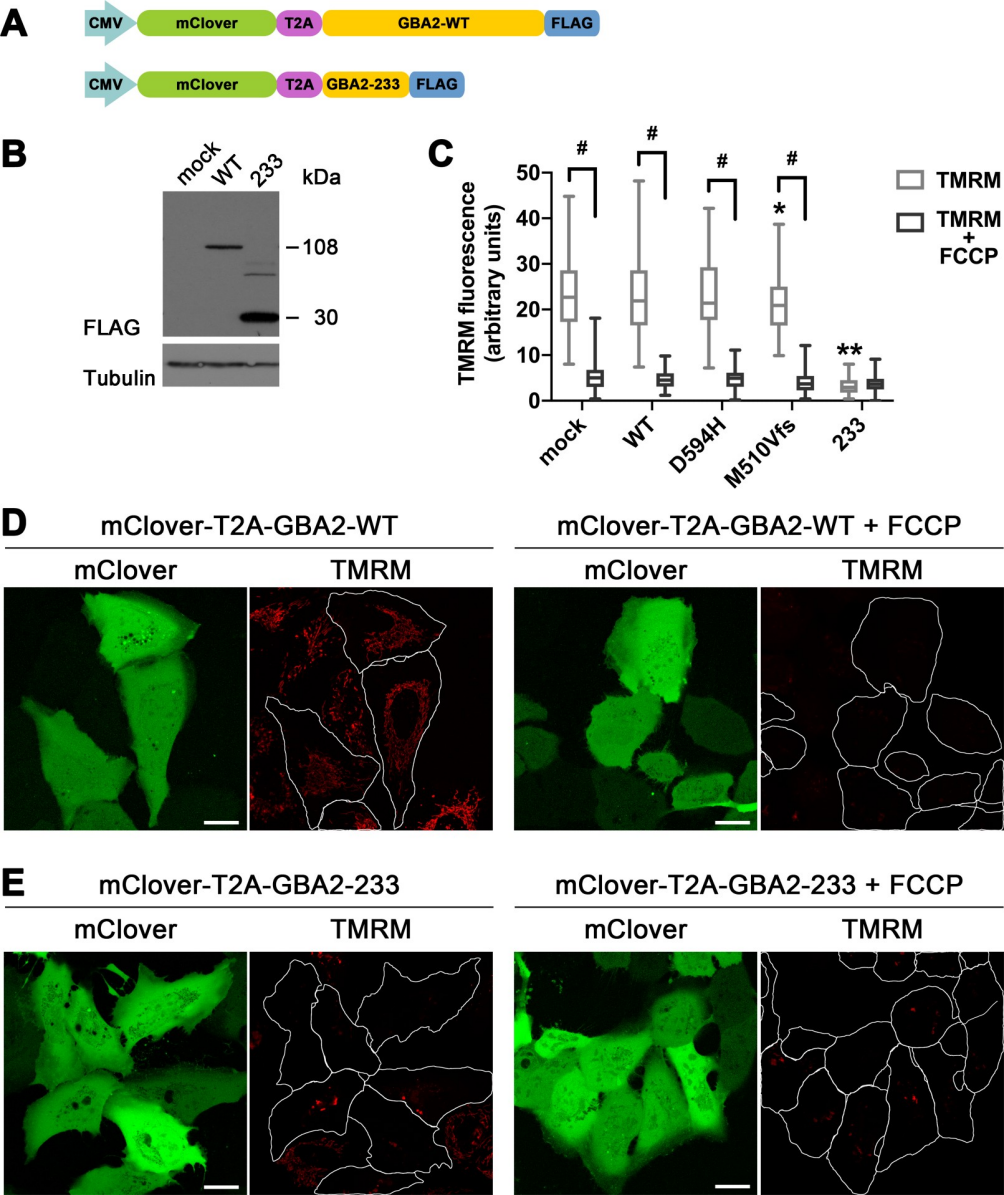
GBA2-233 depletes the mitochondrial transmembrane potential. (A) Schematics of cDNA constructs allowing the co-expression of mClover and WT or GBA2-233. Similar constructs were used for the co-expression of mClover and GBA2-D594, GBA2-M510Vfs*17. (B) Western blot prepared with lysates from U2OS cells transfected with the constructs depicted in [A] probed with an anti-FLAG antibody; apparent molecular weights are indicated. (C) Quantification of transmembrane potential in non-transfected cells and in cells expressing GBA2-WT, GBA2-D594, GBA2-M510Vfs*17, and GBA2-233 (30 cells were analyzed for each of three independent experiments; whiskers, 1–99 percentile). Fluorescence intensity was quantified after exposure of cells to TMRM. Statistical comparisons for the impact of FCCP, using one-way nested ANOVA: #, P < 0.0001. Statistical comparisons with GBA2-WT: *, P = 0.0488; **, P < 0.0001. (D) Non-transfected cells and cells co-expressing mClover (green) and GBA2-WT were imaged live by confocal microscopy after exposure to TMRM (red) (left two panels) or TMRM and FCCP (right two panels). (E) Non-transfected cells and cells co-expressing mClover (green) and GBA2-233 were imaged live by confocal microscopy after exposure to TMRM (red) (left two panels) or TMRM and FCCP (right two panels). Scale bar, 20 μm.

Considering that the mitochondrial transmembrane potential is a prerequisite for the ability of mitochondria to fuse [36], we evaluated whether the mitochondrial fragmentation described above was associated with a reduction in the transmembrane potential. To this end, we used cDNA constructs coding for mClover2, a green fluorescent protein, in tandem with either GBA2-WT or a disease-associated GBA2 mutant, separated by the ribosome-skipping T2A sequence (Fig 6A). To assess the mitochondrial transmembrane potential, we used the fluorescent indicator tetramethylrhodamine methyl ester (TMRM), which is a lipophilic compound carrying a delocalized positive charge. TMRM is therefore membrane-permeant, enters live cells, and distributes across cell membranes in a voltage-dependent manner, accumulating in negatively charged mitochondria in proportion to the potential across the inner membrane [37–39]. Non-transfected cells and cells expressing mClover2 together with GBA2-WT, GBA2-D594, or GBA2-M510Vfs*17 accumulated TMRM in their mitochondria to similar levels (Fig 6C and 6D, left panels), which was prevented by co-incubating the cells with the mitochondrial uncoupling agent carbonyl cyanide 4-(trifluoromethoxy)phenylhydrazone (FCCP) (Fig 6C and 6D, right panels). By contrast, cells expressing mClover2 and GBA2-233 accumulated TMRM at much lower levels, comparable to cells co-incubated with FCCP (Fig 6C and 6E). These results show that GBA2-233 caused the mitochondria to lose their transmembrane potential.

### Mitochondria-fragmenting domain in GBA2

**Fig 7:**
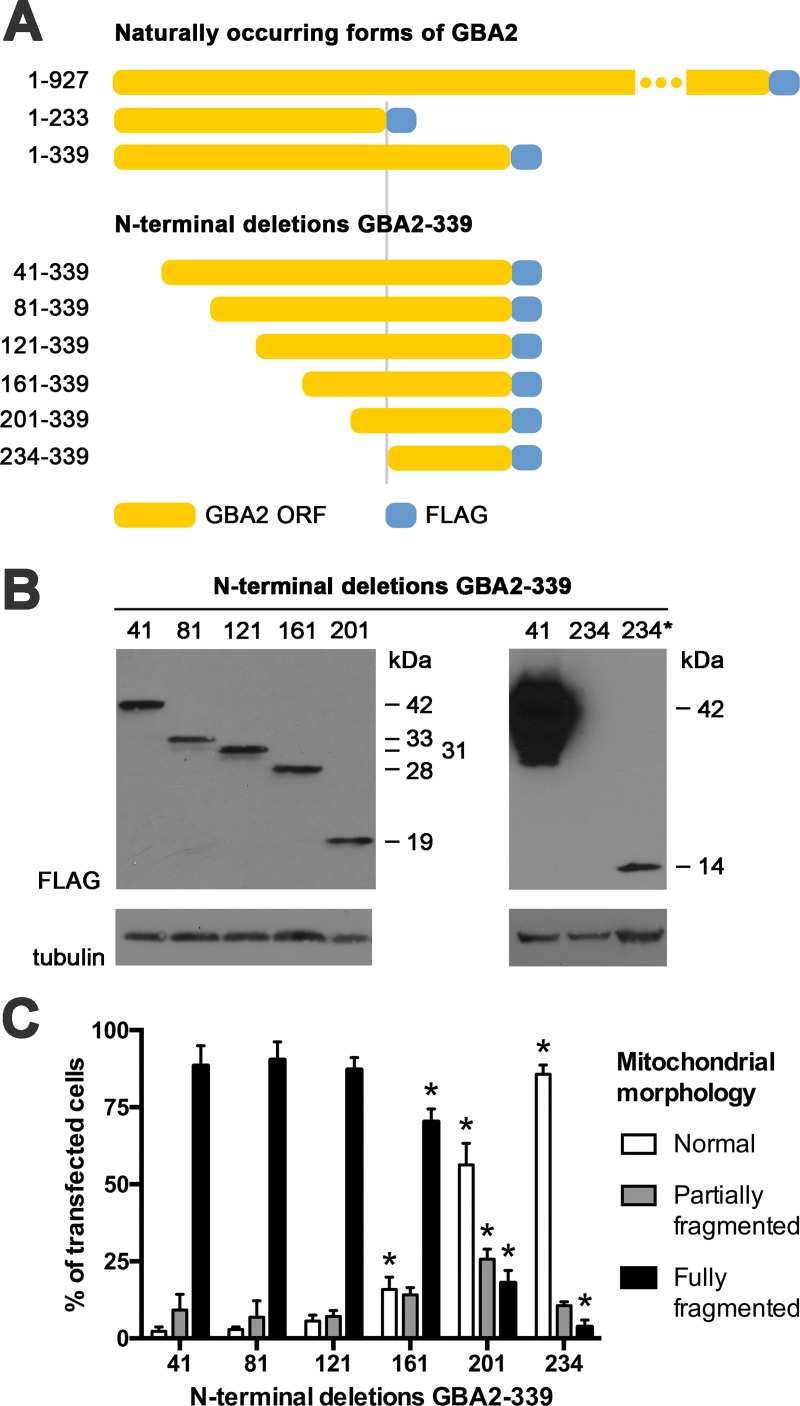
Identification of a mitochondrial targeting domain in GBA2. (A) Schematics of mutant versions of GBA2-339-FLAG carrying increasingly large N-terminal deletions, lacking 40, 80, 120, 160, 200, and 233 amino acids (GBA2[41–339] to GBA2[234–339]); schematics of GBA2-WT, GBA2-233, and GBA2-339 are depicted for reference (schematics drawn to scale, except GBA2-WT). (B) Western blot showing expression of N-terminal deletion mutants depicted in [A] in U2OS cells, “41” indicating GBA2[41–339], etc. Left panel, 8% polyacrylamide gel, 12 μg protein per lane. Right panel, 12.5% polyacrylamide gel; “41” and “234”, 10 μg protein; “234*”, 30 μg protein. Apparent molecular weights are indicated. (C) Distribution of mitochondrial phenotypes in cells expressing different N-terminal deletion mutants of GBA2-339. Data indicate means + SD; at least 250 cells were scored in each of three independent experiments. Asterisk: significantly different compared to cells expressing GBA2[41–339], determined via one-way ANOVA.

**Fig 8:**
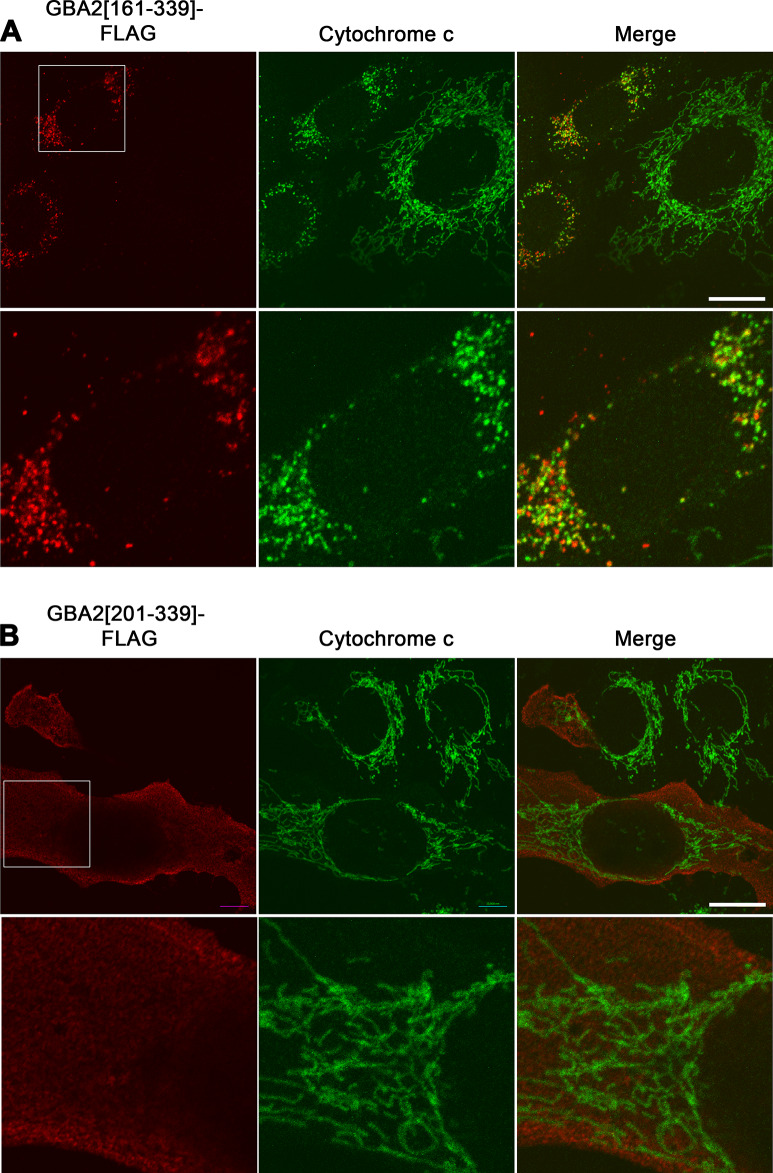
Removal of amino acids 160–200 prevents mitochondrial localization of GBA2-339. U2OS cells transfected with cDNAs coding for (A) GBA2[161–339]-FLAG or (B) GBA2[201–339]-FLAG were immunostained with anti-FLAG (red) and anti-cytochrome c antibodies (green). A section of the images in the upper panels is enlarged in the lower panels, indicated by a white square. Scale bar, 20 μm.

To delineate the part of GBA2-233 that is critical for its impact on mitochondrial morphology, we generated a number of mutants of GBA2-339 carrying increasingly large N-terminal deletions in steps of 40 amino acids, up to residue 233 (Fig 7A). These mutants were expressed at comparable levels, except GBA2[234-339], which had a lower level (Fig 7B). GBA2-339 mutants lacking up to 120 N-terminal amino acids (GBA2[121–339]) caused mitochondrial fragmentation (Fig 7C), while the loss of 40 additional amino acids (GBA2[161–339]) slightly diminished these effects (Figs 7C and 8A). Conversely, GBA2-339 mutants lacking 200 or more N-terminal amino acids (GBA2[201–339] and GBA2[234–339]) did not cause fragmentation (Figs 7C and 8B). These results indicate that the part of GBA2-233 that is responsible for mitochondrial fragmentation is located within residues 121–233, with a particular role for residues 161–200.

## Discussion

Previously, we have investigated the enzymology and pharmacology of GBA2-WT *in vitro* and *in vivo* [1, 2, 4]. We have also established that nonsense and missense mutations in the *GBA2* gene associated with cerebellar ataxia/spastic paraplegia abolish most of its enzyme activity [22]. Here we have examined the cell biology of wild-type and mutant forms of GBA2, using confocal and electron microscopy. Our data show that overexpressed GBA2-WT is located at the plasma membrane. The plasma membrane staining we observed by electron microscopy in GBA2-WT-APEX2-transfected cells closely resembles that of cells expressing a plasma membrane-targeted form of APEX2 [33]. Our findings align with those of Boot *et al*. [40], who observed GFP-GBA2 and GBA2-GFP at the plasma membrane of COS-7 cells. Also, many of the fluorescence microscopy images of Körschen *et al*. [41] show a wide distribution of differently-tagged forms of murine Gba2 throughout transfected HEK293 cells (eGFP-Gba2, Gba2-eGFP, and Gba2-HA), partially overlapping with the ER and the Golgi. Further, these authors show a wide distribution of endogenous Gba2-WT in murine hippocampal neurons. By contrast, Yildiz *et al*. [5] show a reticular immunostaining for endogenous Gba2 in COS cells, which would suggest an ER localization. It thus appears that overexpressed GBA2-WT possibly has a different cellular location (plasma membrane) compared to endogenous GBA2-WT (ER). This possibility needs to be addressed.

We have further established that C-terminally truncated GBA2 mutants (GBA2-233 and GBA2-339) are efficiently imported into the mitochondrial matrix, causing mitochondria to lose transmembrane potential and adopt a fragmented morphology. Using the APEX2-tag, we observed at the ultrastructural level that GBA2-233 and GBA2-339 are located in the mitochondrial matrix. Martell *et al*. [34] showed a similar mitochondrial matrix staining in COS-7 cells expressing a form of APEX targeted to the mitochondrial matrix via a canonical N-terminal mitochondrial targeting sequence. By contrast, a protein domain located at the matrix-side of the inner mitochondrial matrix (the mitochondrial calcium uniporter N-terminally tagged with APEX) gave a much more limited DAB staining that left most of the matrix relatively electron-transparent [34].

In addition, we have identified amino acids 160–200 as the domain instrumental for mitochondrial fragmentation in cells overexpressing GBA2-339. We consistently observed that, in cells expressing GBA2-233 and GBA2-339, mitochondrial fragmentation is accompanied by mitochondrial localization of GBA2-233 and GBA2-339. On this basis, it is not unlikely that amino acids 160–200 of GBA2 are also responsible for the mitochondrial localization of GBA2-233 and GBA2-339, harboring an internal mitochondria-targeting domain. Internal targeting sequences have previously been identified in a small number mitochondrial matrix proteins [42] and can be activated in various ways [43], raising the possibility that GBA2-WT might–under certain conditions–be imported into the mitochondrial matrix. This possibility remains to be experimentally addressed, but would align with an earlier study identifying rat Gba2 in the proteome of brain mitochondria [44]. Moreover, GBA2 tagged with green fluorescent protein co-purified with six mitochondrial proteins, including the matrix protease AFG3L2 and the mitochondrial ribosomal proteins MRPL44 and MRPS22 [45, 46]. Mutations in the genes coding for AFG3L2 and its binding partner paraplegin are associated with a form of spinocerebellar ataxia (SCA28) [47] and a form of hereditary spastic paraplegia (SPG7) [48], respectively.

Compared to human GBA2, the localization of murine Gba2 has not been investigated at the ultrastructural level, so it is not known whether a minority of murine Gba2 is present in mitochondria. However, C-terminally truncated forms of murine Gba2 corresponding to GBA2-233 and GBA2-339 are not localized in mitochondria, but are widely distributed throughout the cell, similar to Gba2-WT and GBA2-WT [17]. Our results thus show that C-terminally truncated mutants of human GBA2 behave differently compared to corresponding mutants of murine Gba2. The basis of this disparity remains to be established. Alternatively, considering that the effects of pharmacologically inhibiting Gba2 in inbred mice greatly depend on their genetic background [14, 15], the possibility remains that murine Gba2 has a more pronounced mitochondrial role in mice with a genetic background distinct from that of the Gba2-deficient mice studied thus far.

Finally, the question is to what extent our observations apply to SPG46 and MSS patients. Considering that GBA2-233 and GBA2-339 cause loss of mitochondrial transmembrane potential and mitochondrial fragmentation in cultured cells, it is not likely that this also happens in patient cells. Persistent mitochondrial fragmentation and subsequent lack of ATP and of neuronal transmembrane potential are incompatible with neuronal activity and may be non-viable. It is more plausible that, in SPG46 patients homozygous for the c.700C>T (p.R234*) and c.1018C>T (p.R340*) mutations, the expression of GBA2-233 and GBA2-339 is attenuated through nonsense-mediated decay, a process that recognizes mRNAs carrying premature stop codons, targeting them for degradation [49]. Altogether, the pathology of these patients is likely due to a deficit of the activities of GBA2-WT, similar to patients carrying other mutations in *GBA2*.

## Supporting information

S1 Fig The GBA2-233 truncation mutant localizes to fragmented mitochondria in Hela, SH-SY5Y, and primary rat hippocampal cells. HeLa cells (A and B), SH-SY5Y cells (C and D) and rat cells (E and F) were transiently transfected with cDNAs coding for GBA2-WT (A, C, and E) or GBA2-233 (B, D, and F), all with a C-terminal FLAG epitope tag. In all three cell types, GBA-WT displayed a plasma membrane distribution. Cells expressing GBA2-WT, as well as non-transfected cells, displayed an extended mitochondrial network, consisting of longer and shorter mitochondria. In contrast, GBA2-233 had a punctate distribution in all three cell types and localized to mitochondria that were very short and irregular in shape. Image panels show whole cells (upper rows) with white squares indicating which areas are shown in greater detail in lower rows. GBA2-WT and GBA2-233 were visualized with anti-FLAG antibodies (red), and mitochondria with anti-TOMM20 (green). Scale bar: 20 mm.
(TIF)

S2 Fig Localization of APEX2-tagged GBA2-WT and -233 via proximal protein biotinylation. U2OS cells transfected with cDNA constructs coding for (A) GBA2-WT-APEX2 and (B) GBA2-233-APEX2 were incubated with biotin-phenol and briefly exposed to hydrogen peroxide, which activates the peroxidase activity of APEX2. Biotinylated proteins were detected with Alexa594-conjugated streptavidin (red) while mitochondria were stained with anti-TOMM20 (green). Scale bar, 20 μm.
(TIF)

S3 Fig GBA2-D594H-FLAG and TST-GBA2-M510Vfs*17 are distributed throughout the cell. (A) U2OS cells transfected with a cDNA coding for GBA2-D594H-FLAG were immunostained with anti-FLAG (red) and anti-TOMM20 antibodies (green). (B) U2OS cells transfected with a cDNA coding for TST-GBA2-M510Vfs*17 were immunostained with anti-TST (red) and anti-TOMM20 antibodies (green). A section (white square) of the images in the upper panels is enlarged in the lower panels. Scale bar, 20 μm.
(TIF)

S4 Fig When expressed under the control of the MSCV LTR, GBA2-233-FLAG localizes to fragmented mitochondria. U2OS cells were transfected with a cDNA coding for (A) GBA2-WT-FLAG and (B) GBA2-233-FLAG under the control of the MSCV LTR, and immunostained with anti-FLAG (red) and anti-cytochrome c antibodies (green). A section (white square) of the images in the upper panels is enlarged in the lower panels. Scale bar, 20 μm.
(TIF)

S1 Raw Images (PDF)
