# Supplementary figures and images for: Truncated mutants of beta-glucosidase 2 (GBA2) are localized in the mitochondrial matrix and cause mitochondrial fragmentation
Source: PLoS One. 2020 Jun 3;15(6):e0233856. doi: 10.1371/journal.pone.0233856 (PMC7269613; doi:10.1371/journal.pone.0233856)

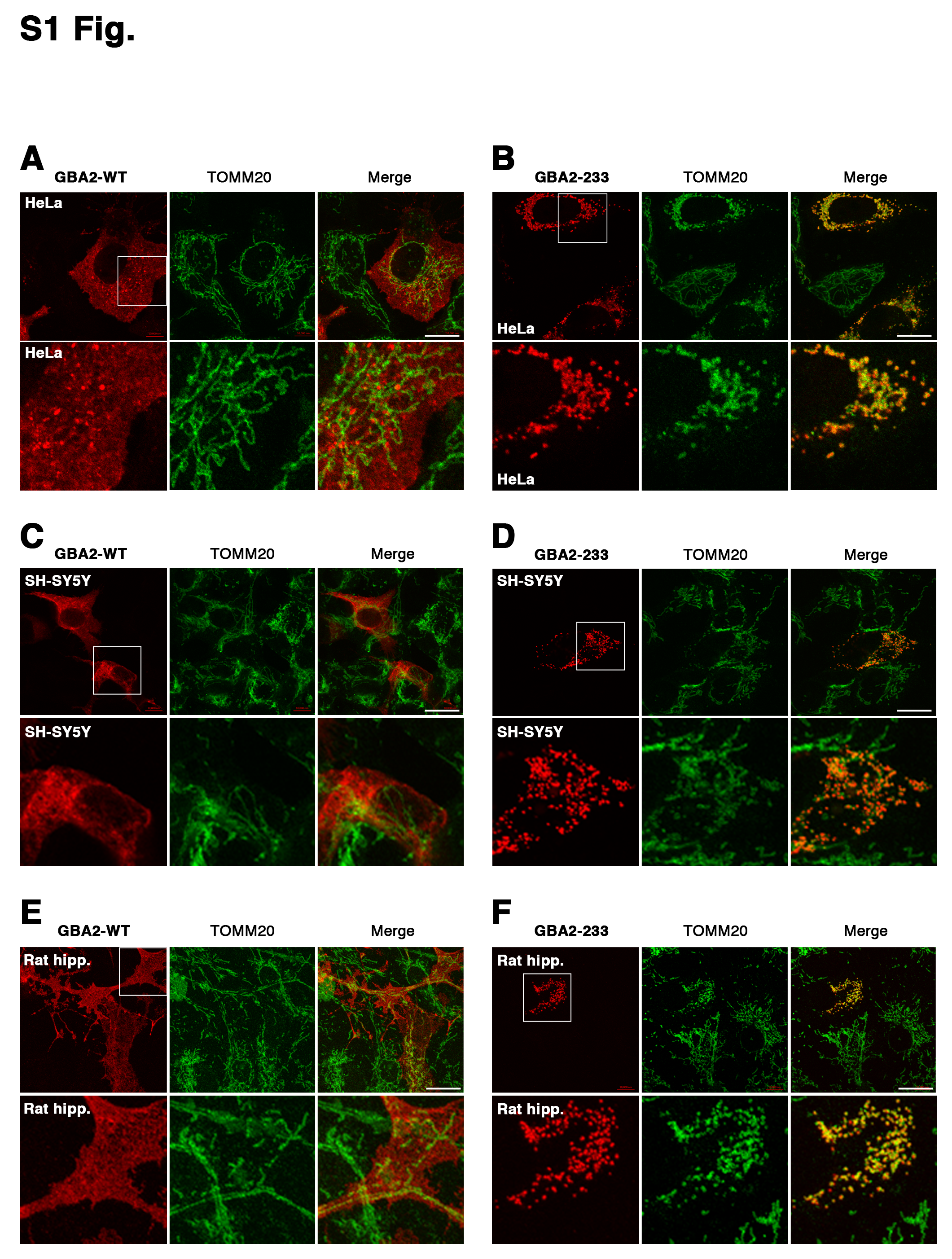

Supplement: S1 Fig — HeLa cells (A and B), SH-SY5Y cells (C and D) and rat cells (E and F) were transiently transfected with cDNAs coding for GBA2-WT (A, C, and E) or GBA2-233 (B, D, and F), all with a C-terminal FLAG epitope tag. In all three cell types, GBA-WT displayed a plasma membrane distribution. Cells expressing GBA2-WT, as well as non-transfected cells, displayed an extended mitochondrial network, consisting of longer and shorter mitochondria. In contrast, GBA2-233 had a punctate distribution in all three cell types and localized to mitochondria that were very short and irregular in shape. Image panels show whole cells (upper rows) with white squares indicating which areas are shown in greater detail in lower rows. GBA2-WT and GBA2-233 were visualized with anti-FLAG antibodies (red), and mitochondria with anti-TOMM20 (green). Scale bar: 20 mm. (TIF) [file pone.0233856.s001.tif]

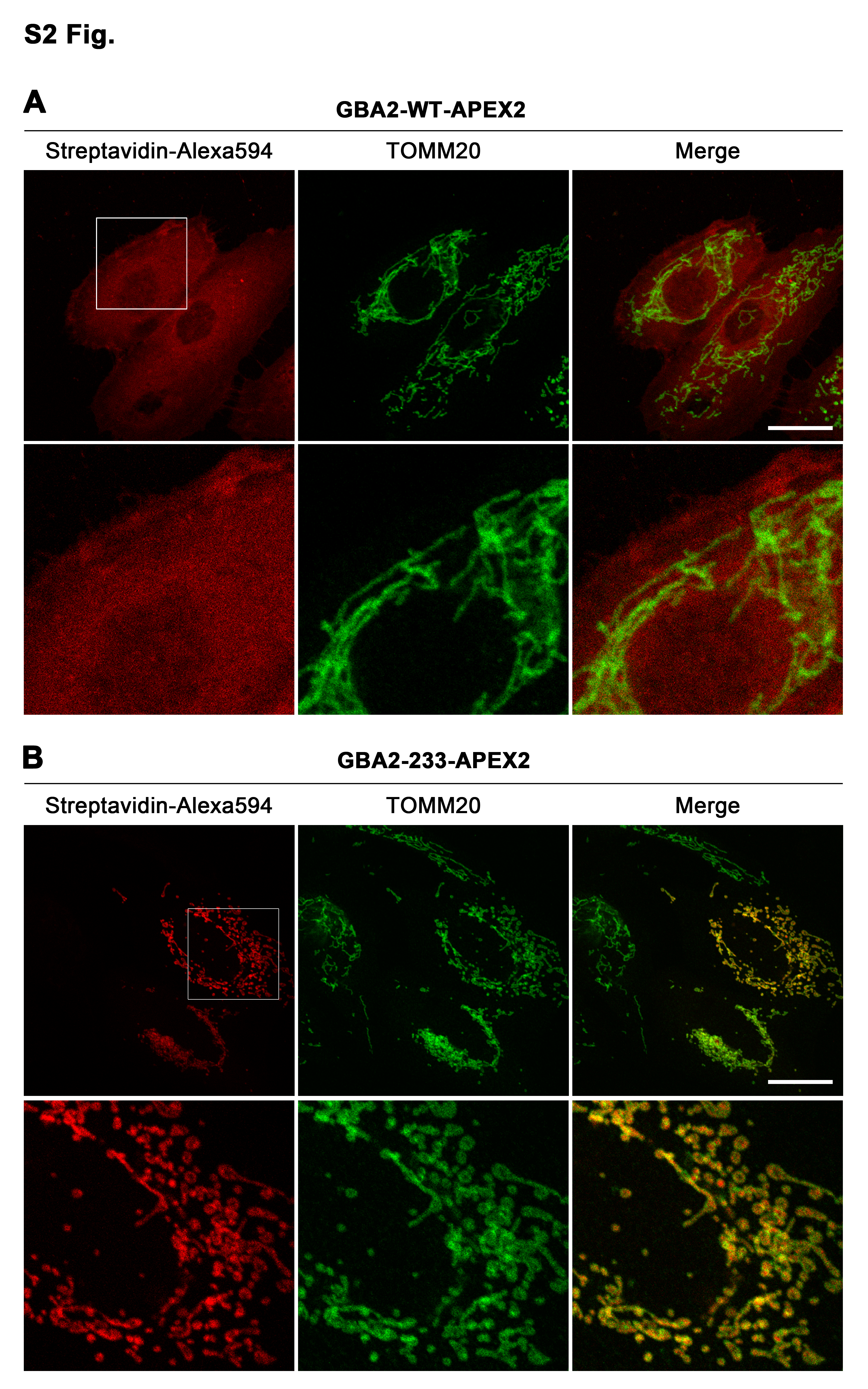

Supplement: S2 Fig — U2OS cells transfected with cDNA constructs coding for (A) GBA2-WT-APEX2 and (B) GBA2-233-APEX2 were incubated with biotin-phenol and briefly exposed to hydrogen peroxide, which activates the peroxidase activity of APEX2. Biotinylated proteins were detected with Alexa594-conjugated streptavidin (red) while mitochondria were stained with anti-TOMM20 (green). Scale bar, 20 μm. (TIF) [file pone.0233856.s002.tif]

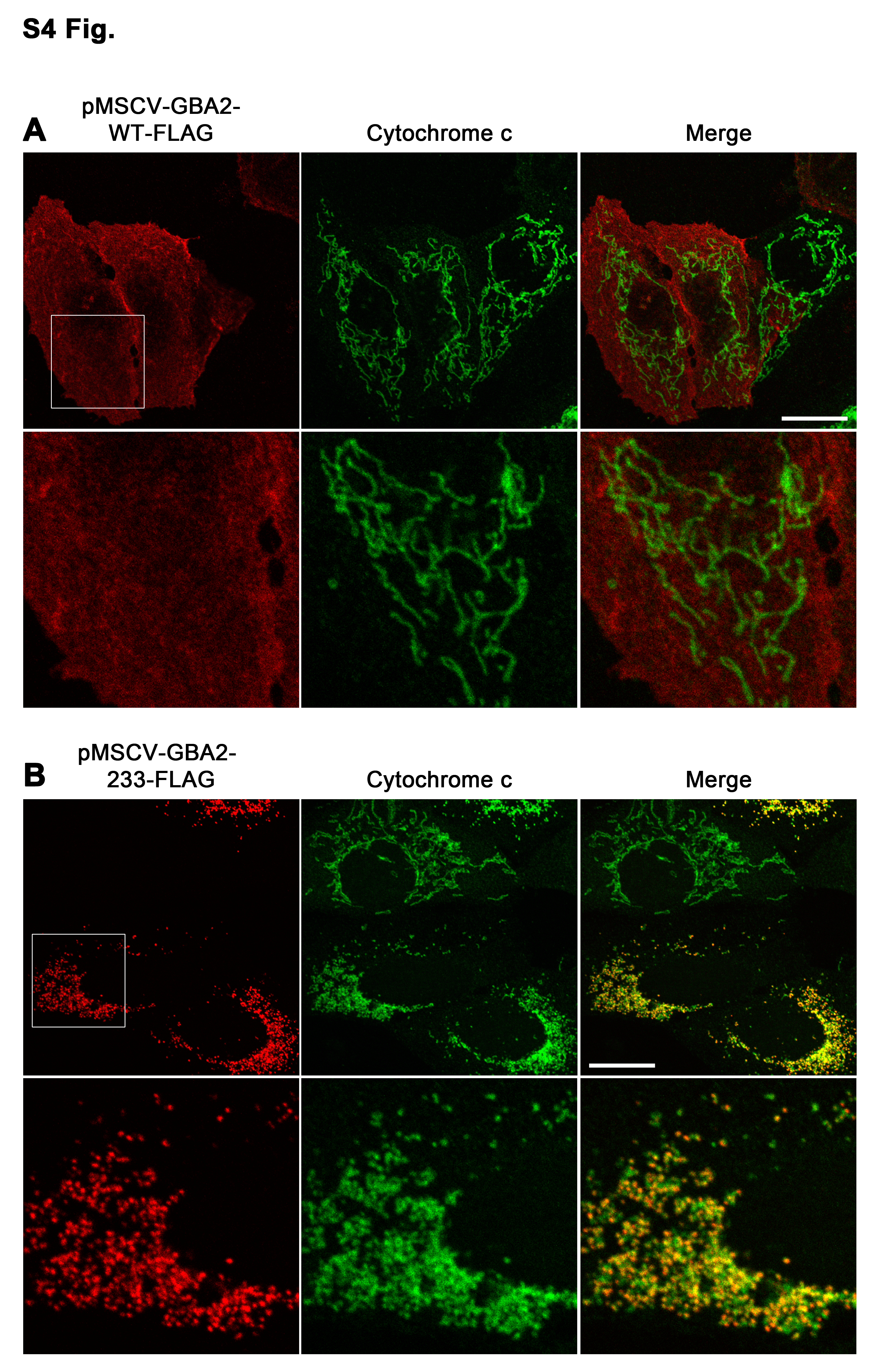

Supplement: S4 Fig — U2OS cells were transfected with a cDNA coding for (A) GBA2-WT-FLAG and (B) GBA2-233-FLAG under the control of the MSCV LTR, and immunostained with anti-FLAG (red) and anti-cytochrome c antibodies (green). A section (white square) of the images in the upper panels is enlarged in the lower panels. Scale bar, 20 μm. (TIF) [file pone.0233856.s004.tif]
