# Supplementary material for: Truncated mutants of beta-glucosidase 2 (GBA2) are localized in the mitochondrial matrix and cause mitochondrial fragmentation
Source: PLoS One. 2020 Jun 3;15(6):e0233856. doi: 10.1371/journal.pone.0233856 (PMC7269613; doi:10.1371/journal.pone.0233856)
Supplement: S1 Raw Images — (PDF) [file pone.0233856.s005.pdf]

Fig 1 E (top panel)

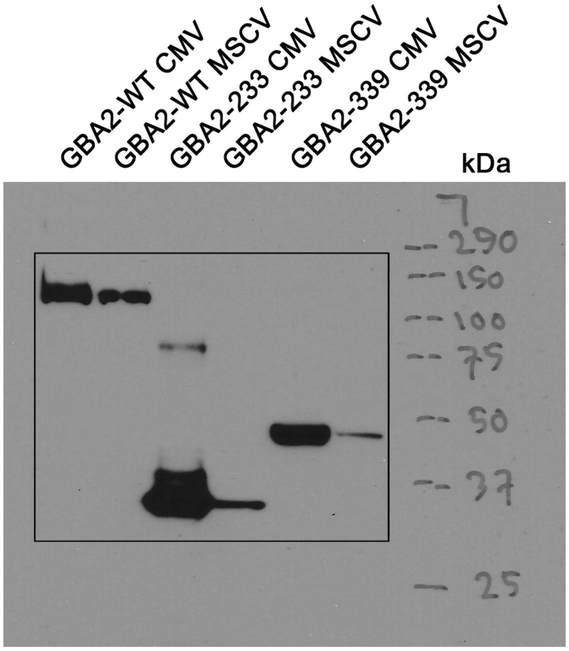

Anti-FLAG antibody; chemiluminescent substrate; X-ray film.

Fig 1 E GAPDH loading control

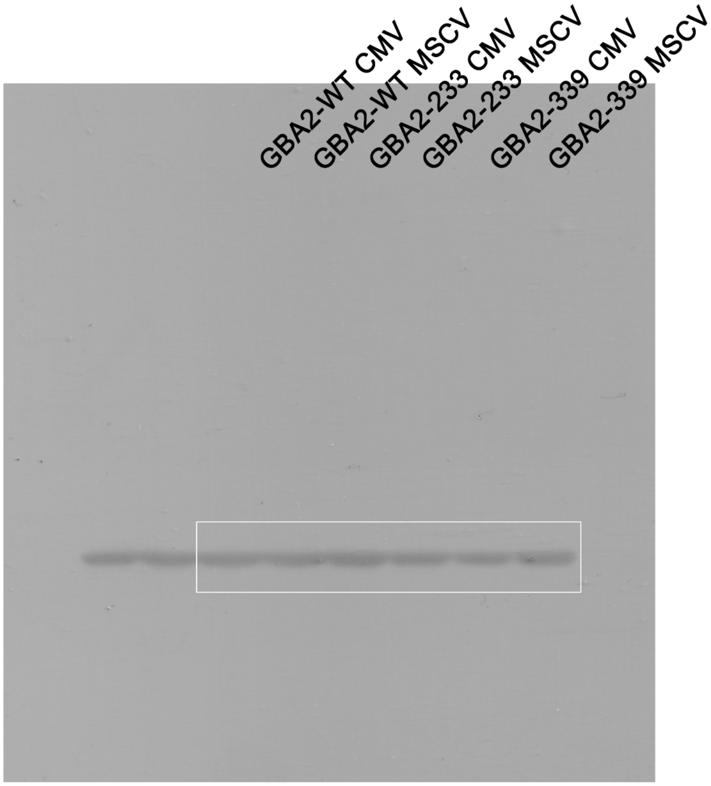

Anti-GAPDH antibody; chemiluminescent substrate; X-ray film.

Fig 1 F

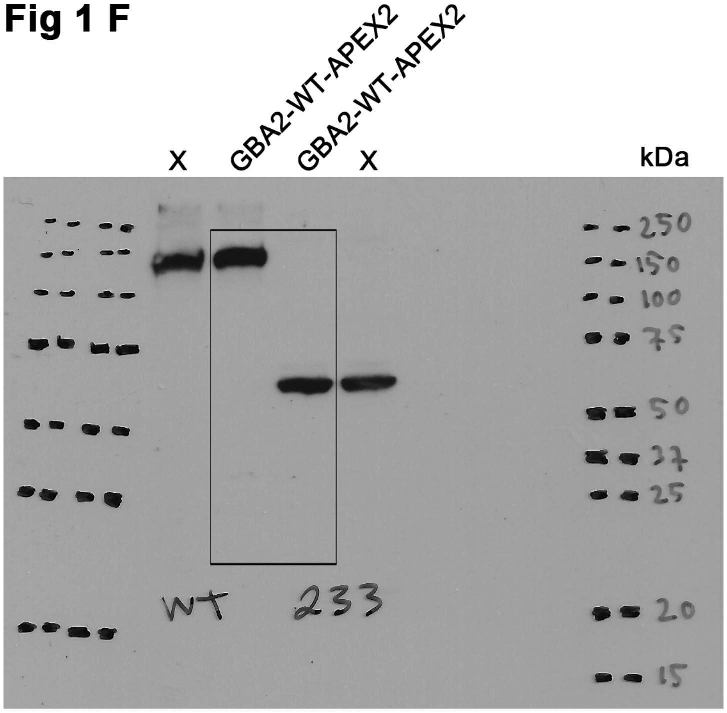

Anti-HA antibody; chemiluminescent substrate; X-ray film.

Fig 6 B (upper panel)

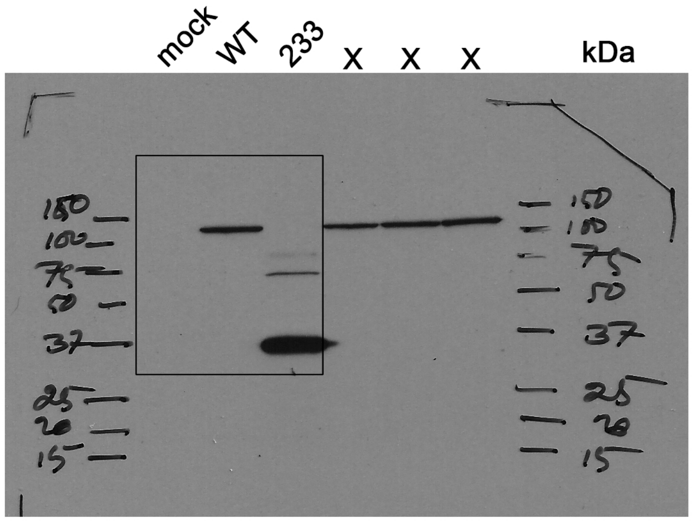

Anti-FLAG antibody; chemiluminescent substrate; X-ray film.

Fig 6 B (loading control)

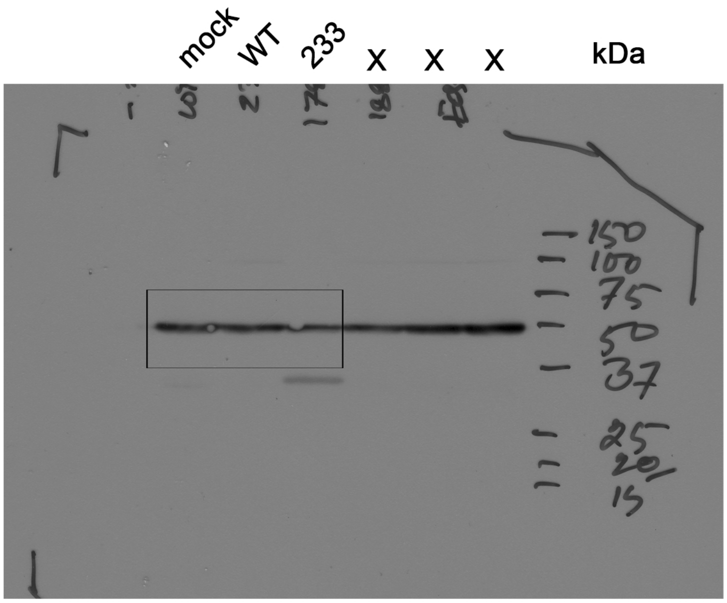

Anti-tubulin antibody; chemiluminescent substrate; X-ray film.

Fig 7B Upper left panel

41 81 121 161 201 X X kDa

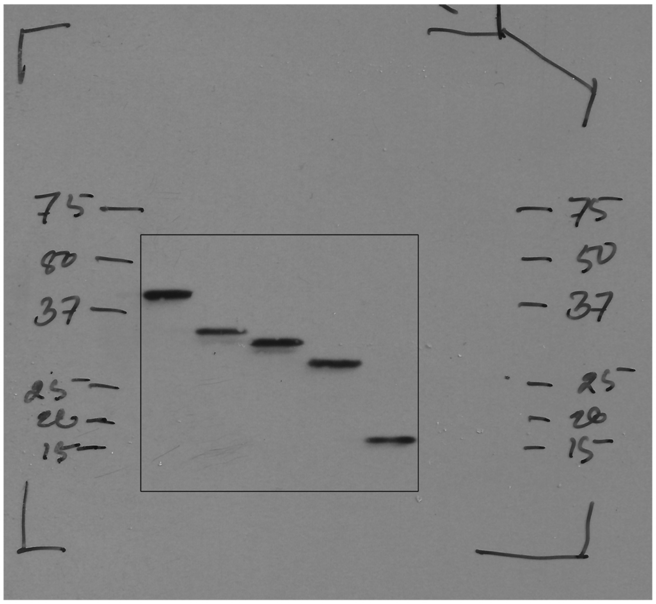

anti-FLAG; chemiluminiescent substrate; X-ray film.

Fig 7B Lower left panel (loading control)

41 81 121 161 201 X X kDa

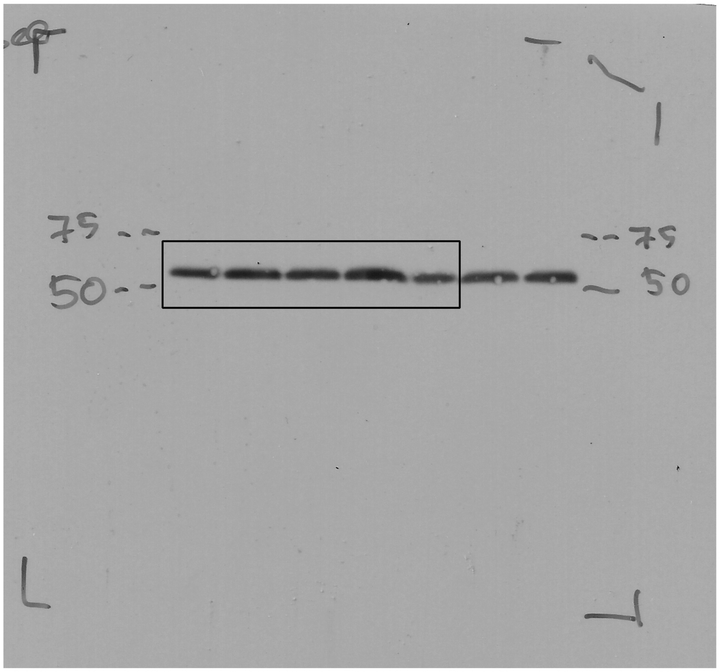

anti-tubulin; chemiluminiescent substrate; X-ray film.

Fig 7 B Upper right panel

41 234 234* kDa

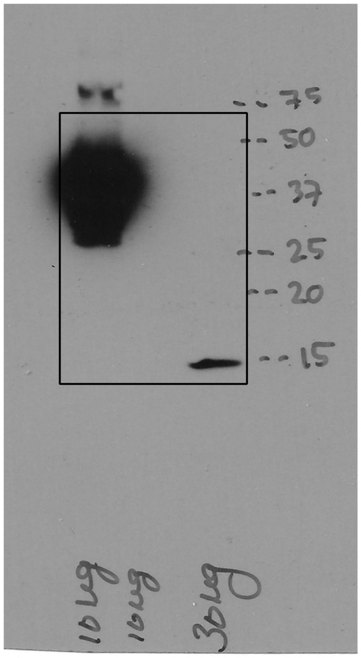

anti-FLAG; chemiluminescent substrate; X-ray film.

Fig 7 B Lower right panel - Loading control

41 234 234* kDa

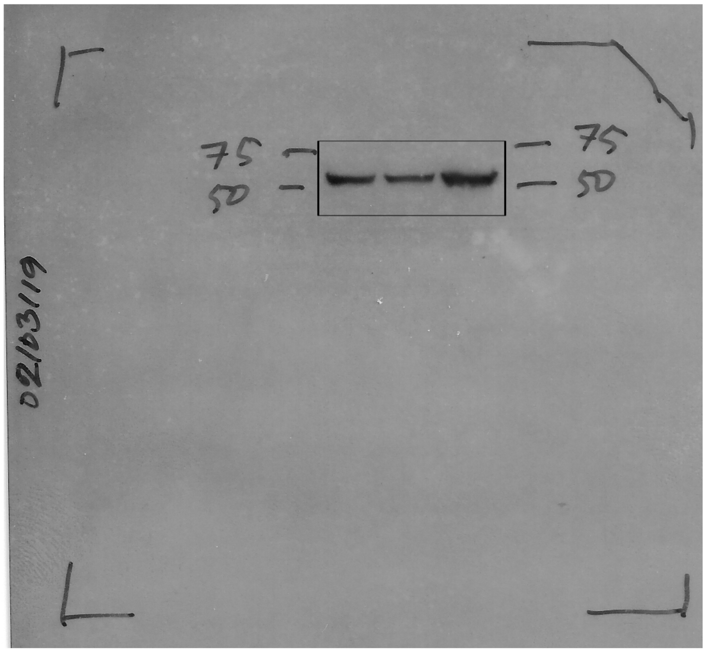

anti-tubulin; chemiluminescent substrate; X-ray film.
